# Single-item Patient Enablement Instrument (PEI-Q2) as a feedback tool in primary health care: a qualitative study among Finnish primary care physicians

**DOI:** 10.3399/BJGPO.2025.0126

**Published:** 2026-05-20

**Authors:** Ida Tiihonen, Tuomas H Koskela, Aapo Tahkola, Elina Tolvanen

**Affiliations:** 1 Faculty of Medicine and Health Technology, Tampere University, Tampere, Finland; 2 Wellbeing Services County of Central Finland, Jyväskylä, Finland; 3 Wellbeing Services County of Pirkanmaa, Pirkanmaa, Finland; 4 Finnish Institute for Health and Welfare, Helsinki, Finland

**Keywords:** primary health care, general practitioners, patient feedback, patient enablement, quality of health care, qualitative research

## Abstract

**Background:**

In Finland, the gathering of patient feedback using patient-reported outcome measures, such as the Patient Enablement Instrument (PEI), is recommended to assess the quality of care. However, knowledge of how this feedback could be utilised by the individual primary care physician (PCP) is sparse.

**Aim:**

To explore PCPs’ experiences of the single-item PEI (PEI-Q2) as a feedback tool.

**Design & setting:**

Qualitative study in Finnish primary health care.

**Method:**

Semi-structured focus group interviews were conducted with 14 PCPs in June 2023 in Central Finland after they had received feedback with the PEI-Q2. The data were analysed using thematic analysis.

**Results:**

PCPs regarded patient enablement as an important indicator of care quality in primary health care. The feedback provided through the PEI-Q2 was generally perceived as encouraging and motivating, prompting requests for its regular use. However, several PCPs found the feedback challenging to interpret. Feedback with the PEI-Q2 was seen as a promising tool for managerial benchmarking. Additionally, PCPs suggested that the utility of the PEI-Q2 feedback could be enhanced by incorporating supplementary contextual information such as open-ended feedback.

**Conclusion:**

From the PCPs’ viewpoint, the PEI-Q2 appears to be a potential tool for delivering patient feedback. While it can serve as a measure for assessing quality of care, it should be complemented with other patient-reported feedback tools to enhance its overall applicability and usefulness. In addition, educational guidance on interpreting the measure’s results is recommended.

## How this fits in

In patient-centred care, it is essential to consider the perspectives of patients regarding the care they receive. In Finland, primary healthcare systems are currently implementing patient-reported quality indicators. One potential way to measure patients’ views is the Patient Enablement Instrument (PEI) and its single-item version, the PEI-Q2. Previous studies lack information on how this measure could be utilised as feedback for primary care physicians. Using thematic analysis, this study examined primary care physicians’ initial perceptions of the PEI-Q2 as feedback instrument. The findings indicated that participants were receptive to receiving patient feedback based on the PEI-Q2, especially in combination with other patient-reported outcome measures.

## Introduction

Physicians need feedback from their patients to improve their performance. However, physicians are poor at predicting the feedback their patients give.^1^ Therefore, without feedback, the patients’ views of the care they receive may remain unknown. In a recent study, almost half of primary care physicians (PCPs) expressed interest in receiving systematically collected patient feedback.^2^ Nevertheless, it is suggested that individual PCPs rarely use patient experience feedback for quality improvement.^3^

Patient feedback is associated with PCPs’ enhanced interpersonal skill development^4^ and improved medical performance.^5^ However, the impact of patient feedback may not only be positive. In certain circumstances, it can lead to increased stress, decreased job satisfaction, and inappropriate medical decisions.^6,7^ The acceptance of feedback is significantly influenced by the methods used for its collection and delivery. Feedback that is specific, collected through credible methods, and includes narrative information is more likely to be impactful.^5^

Patient-reported outcome measures (PROMs) and patient-reported experience measures (PREMs) are ways to measure patients’ views. PROMs are standardised measures aiming to fill the gap between physicians’ and patients’ views of the clinical reality. PROMs can be used for quality improvement, benchmarking, and performance assessment.^8^ PREMs give insights into patients’ experiences of care and can also be used to drive quality improvement.^9^ In Finland, the Finnish Institute for Health and Welfare suggests the same measures for national use for quality follow-up.^10^ One example of a PREM is the Net Promoter Score (NPS), which assesses the likelihood that users would recommend the service to a friend or colleague.^11^

One of the potential PROMs for primary care is the PEI.^12^ The PEI measures patient enablement, how patients perceive their ability to cope with their illness and life following non-urgent consultations with a GP.^12^ A previous study has shown that the PEI is suitable for the Finnish primary healthcare population.^13^ Originally, the PEI instrument consisted of six items. A single-item version of the PEI (PEI-Q2) has been shown to identify patients who give low scores in the whole questionnaire.^14^

Several healthcare organisations in Finland, including public wellbeing services counties and some private organisations, have implemented the PEI-Q2 (Elina Tolvanen, personal correspondence, April 2025). However, we lack knowledge about the professionals’ perceptions. The acceptance of the PEI among physicians has been studied previously in one study.^15^ To our knowledge, there are no previous studies that have explored PCPs’ views or experiences regarding the PEI-Q2. As patient-reported feedback data accumulates, it is crucial to consider how these data can be utilised on administrative, managerial, and professional levels. In our study, we aimed to explore primary care physicians’ perceptions of the PEI-Q2 as a feedback tool.

## Method

This was a qualitative study using thematic analysis. We conducted semi-structured focus group interviews with PCPs, discussing their experiences and perspectives on the PEI-Q2.

All the researchers were involved in designing the study and interpreting the data: IT (GP registrar); ET (GP, PhD); AT (GP, PhD); and TK (GP, professor of general practice). IT had prior contact with most of the study participants by working at the same unit in the Wellbeing Services County of Central Finland as a GP registrar. IT has no previous experience of the PEI or qualitative research, but she has participated in a methodology course on qualitative research. ET conducted her PhD thesis on the patient enablement measurement under the supervision of TK. ET has participated in several qualitative studies and participated in a methodology course on qualitative research. AT has worked with the study participants previously. AT has no previous experience of the PEI or qualitative research, but he is currently working for the Finnish Institute for Health and Welfare as a clinical director of effectiveness on similar topics. TK has previous experience conducting qualitative research and has contributed to numerous projects.

Our study was conducted in the Wellbeing Services County of Central Finland in spring 2023. This qualitative study was a part of a larger, ongoing study that aimed to determine if physicians who received PEI feedback and brief training about patient enablement could improve their patients’ PEI results. Based on power calculation, the target number of participating PCPs was 20.

All PCPs working at nine health centres were invited to participate in the study. Reminders were sent until the target of 20 participants was reached. The participants came from three health centres (two urban, one rural: populations 57 700, 20 700, and 4000, respectively). The participants had no previous knowledge of the PEI-Q2.

The participating PCPs asked their patients to fill in a voluntary, anonymous feedback questionnaire after non-urgent appointments (see Supplementary Information S1). The questionnaire included the PEI-Q2 (*‘As a result of your visit to the doctor today, do you feel you are able to cope with your illness?’* Answering options: Much better [2 points]; Better [1 point]; Same or Less [0 points, respectively]; and, I don’t know [0 points]^12,14^). The Finnish version of PEI-Q2 is provided in Supplementary Information S2. The patients returned the feedback sheets to boxes outside the appointments. The feedback collection period lasted 10 weeks between march and may in 2023.

The PCPs were divided into two groups to participate in an educational event. The purpose of the event was to help participants understand their PEI-Q2 results. In the events, a short lecture about patient enablement was presented by ET or IT, and the PCPs received their patient feedback results. An example of the feedback is presented in Figure 1. The feedback included the participant’s own PEI-Q2 distributions, the distribution of all answers in the follow-up period, and ‘the best’ distribution, meaning the one that had the largest share of patients answering ‘much better’ to the feedback query.

**Figure 1.**
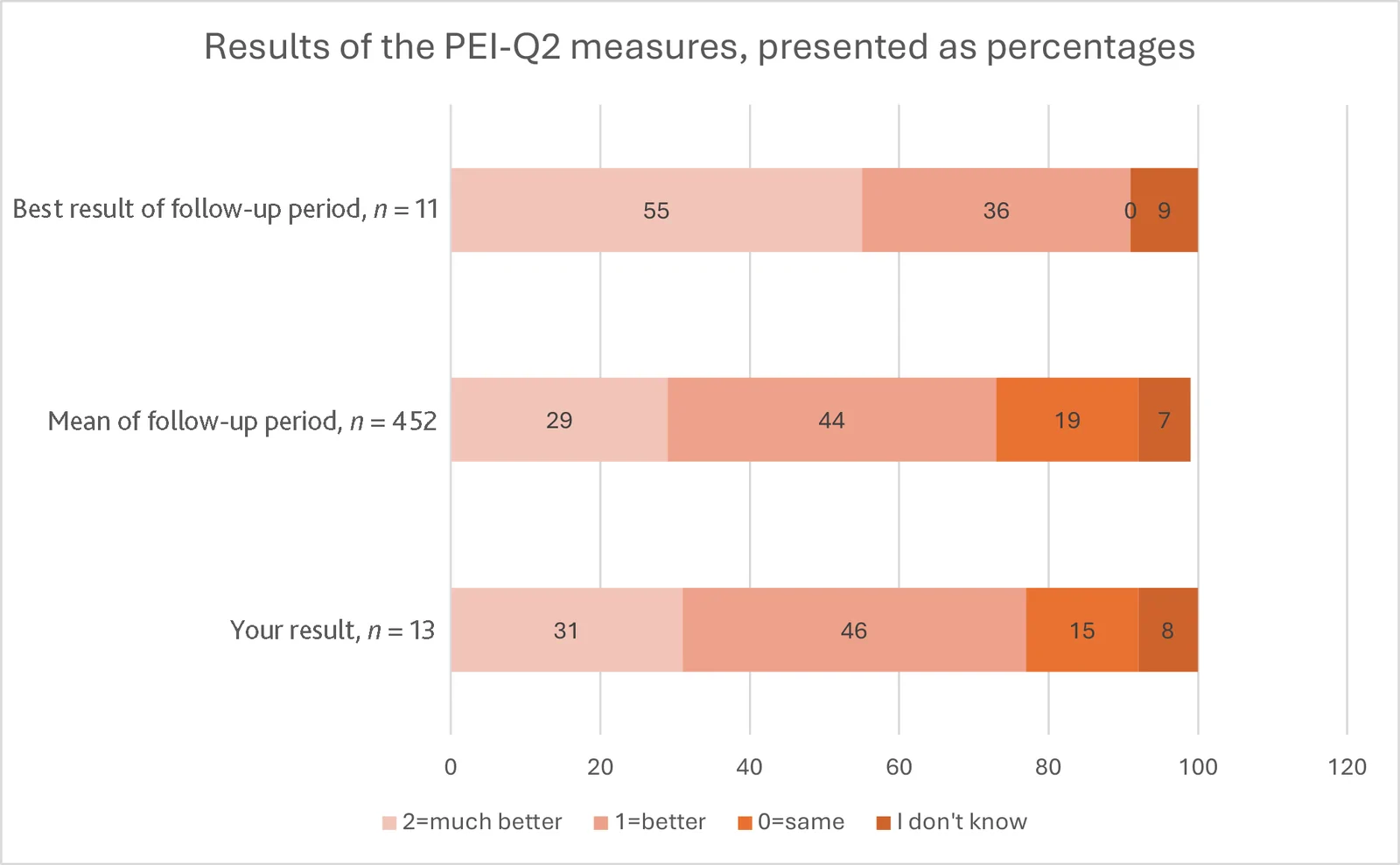
An example of the single-item Patient Enablement Instrument (PEI-Q2) feedback received by a primary care physician at the educational event. PEI-Q2 question: As a result of your visit to the doctor today, do you feel you are able to cope with your illness? Answering options: Much better (2 points); Better (1 point); Same or Less (0 points, respectively); and, I don’t know (0 points). No patients selected the option ‘Less’.

### Data collection

Three semi-structured focus group interviews were performed for 14 participants. Eleven participants attended the interviews face-to-face and three attended online via Microsoft Teams. In each interview, IT was the interviewer, and each co-author acted as an observer via Microsoft Teams. The interviews took place in June 2023 at Nova Health Center, Jyväskylä. The interviews lasted 44–62 minutes and were recorded with consent for transcription. Each interview had four or five participants. An interview guide was used (see Supplementary Information S3). The interview guide was piloted in the first interview, and no changes were made. Open dialogue was emphasised, and the interviewer ensured that all topics in the interview guide were covered.

### Analysis

We used an inductive thematic analysis approach, in which coding was data-driven and not guided by a predefined theoretical framework.^16^ The interviews were initially recorded using Microsoft Teams and subsequently transcribed to Microsoft Word. The analysis followed the established phases of thematic analysis.^16^ First, the research team familiarised themselves with the data. Next, initial coding was conducted by IT and ET, with all codes critically reviewed by the entire research group to ensure consistency and relevance to the study aim.

Following this, similar codes were collated into preliminary categories, which were then reviewed collaboratively. In the subsequent phase, sub-themes, themes, and overarching main themes were identified and named. Throughout the process, interpretations were continuously discussed and cross-checked against the original quotes in the interview transcripts to enhance reflectivity. An example of data are presented in Table 1. Data management and coding were facilitated using Atlas.ti and Microsoft Excel. The analysis was conducted between October 2023 and April 2024.

**Table 1.** An example of data coding, including quotations, corresponding codes, sub-themes, and main themes

Quotation	Code	Sub-theme	Main theme
*‘I'm currently just a licensed physician, but ultimately, I think the core of general medicine is about coping and the patient’s experience of it, as well as understanding the illness and everything that comes with it. So, it’s a good question in principle.’* (A5)	Patient enablement is in the core of general medicine	Patient enablement is essential	Importance of patient enablement
*‘It is absolutely essential, because that’s what I perceive my work to be about — helping patients cope better. The purpose of the appointment is not for the patient to find out how right the doctor is about something, but for them to manage their illness better.’* (B2)	Patient enablement is essential		
*‘It is an essential matter because if the patient copes well, they won't call again in a week. If they cope poorly, they will call again tomorrow and every day after that until they cope better.’* (A2)			
*‘And yes, I also see and feel that gender and age do have an impact. Some people may have a kind of preconceived attitude — like I've been told, “I don't like female doctors“ or “female doctors aren't as competent“, and some have even refused to see a female doctor.’* (A3)	Physician’s age and gender affect the PEI results	Impact of a doctor’s qualities on PEI outcomes	Physician-related factors explaining PEI outcomes
*‘It’s interesting to see whether our age or experience, or our gender, had an impact on the results — for example, whether clearly more experienced people had significantly better outcomes, or whether men had better results due to certain societal expectations.’* (A1)	It is interesting to compare PEI results among physicians of different ages, genders, and levels of experience		
*‘I don’t know if people just have so many bad experiences with health care, unfortunately, but it seems like even if you're just basically polite and make eye contact, they’re like, “Wow, finally a good doctor!“ Even though you’re thinking, “Good grief, I didn’t really do anything except say hello, oh dear, and have a nice day.“ So that kind of skews the feedback a bit.’* (A5)	Patients can already give good feedback simply because of physicians basic manners	Physician behaviour	
*‘I always try to make them feel like it was a good thing they came to the appointment, that their issue is being taken care of, and that they feel heard and respected — even if the matter might conflict with my own values. I want it to feel like the issue has a clear beginning and end.’* (B4)	The feeling of enablement can be influenced by listening and showing appreciation		

A = GP registrar or other physician. B = GP. PEI = Patient Enablement Instrument.

This publication has been prepared in accordance with the Standards for Reporting Qualitative Research guidelines.^17^ Copilot AI was utilised for the translation of article quotations and for language editing purposes.

## Results

The characteristics of the study participants are presented in Table 2. The majority of study participants were female (86%, *n* = 12), and a minority were GPs.

**Table 2.** Characteristics of study participants (*N* = 14)

Characteristic	*n* (%)
**Age, years^a^**	
<30	6 (43)
30–40	4 (29)
>40	4 (29)
**Sex**	
Male	2 (14)
Female	12 (86)
**Level of training**	
Unspecialised physician	4 (29)
GP registrar	6 (43)
GP	4 (29)
**Work experience, years**	
<2	4 (29)
2–6	6 (43)
>6	4 (29)

^a^Median age = 32 years.

The following five main themes emerged from the data: patient enablement as an essential concept; PEI-Q2 as a measure; interpretation of PEI-Q2 feedback; factors influencing PEI-Q2; and the use of PEI-Q2 results as a tool for comparison. In addition, the participants identified future development suggestions for the PEI-Q2. The themes are presented in Figure 2.

**Figure 2.**
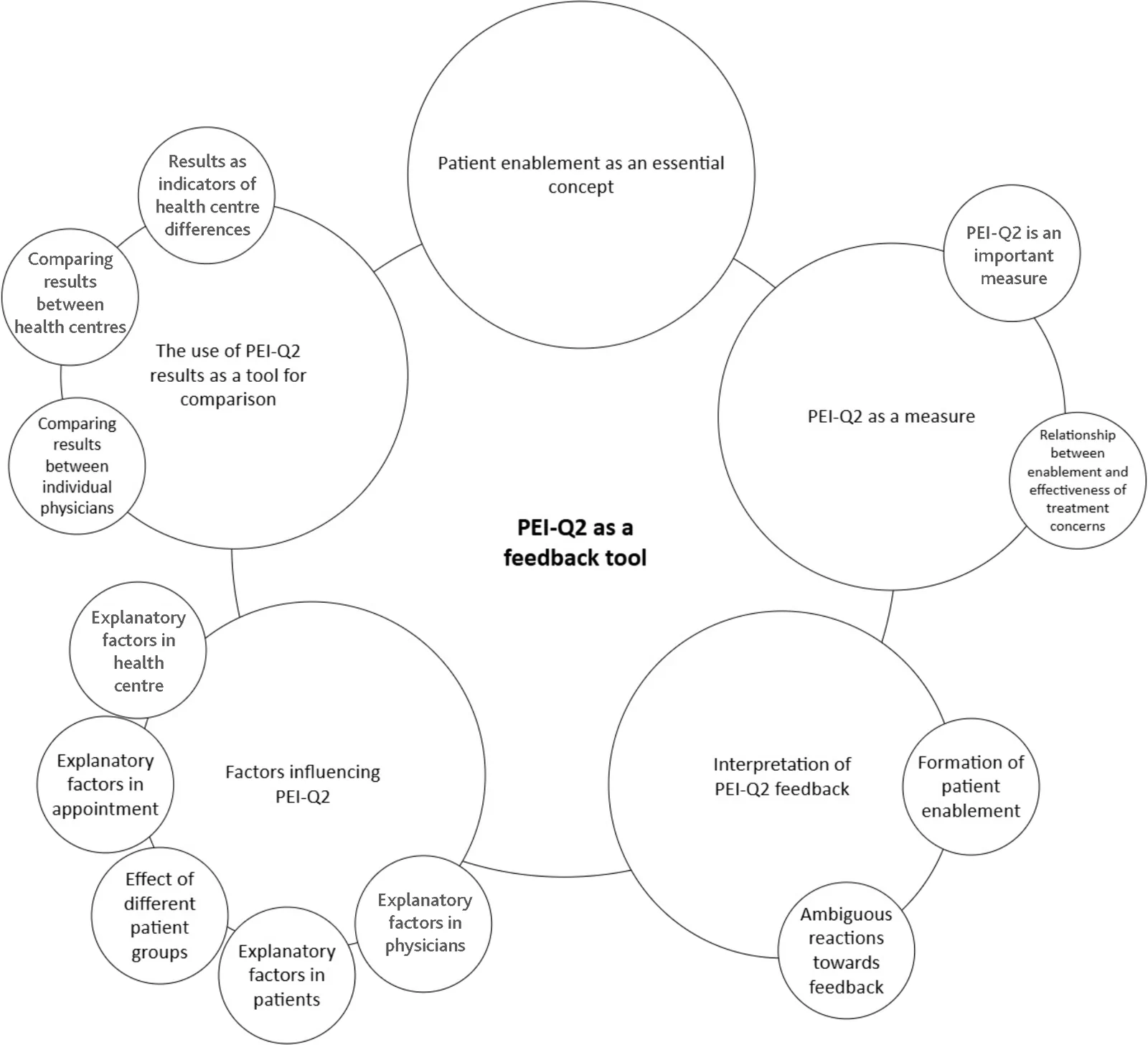
Main themes and sub-themes derived from the data

### Patient enablement as an essential concept

The participants considered patient enablement to be essential in the primary healthcare context. It was recognised as a key concept, and they felt that it should be supported:

*‘Well, it is absolutely essential, because that’s how I see my work, that I help patients to manage better. The purpose of the consultation is not for the patient to find out how right the doctor is about something, but for them to cope better with their illness.’* (B2 [B = GP])

### PEI-Q2 as a measure

Participants regarded the PEI-Q2 as an important measurement tool for their patients. They noted that compared with the NPS, the PEI-Q2 was more comprehensive and better revealed the patient’s perspective on coping with disease. Additionally, the participants expressed a willingness to receive PEI-Q2 feedback regularly. They felt it could encourage and motivate them in their practice. However, some participants had concerns about the relationship between the effectiveness of treatment and patient enablement, whether it represents effectiveness of care or patient satisfaction:

*‘And it really is an important indicator for the patient — how they feel they are coping. From the patient’s perspective, it’s probably one of the most important measures. After all, a patient can't be satisfied if they feel they aren't managing their illness well.’* (A1 [A = GP registrar or other physician])

### Interpretation of PEI-Q2 feedback

Some participants perceived the PEI-Q2 feedback as too generic and difficult to interpret. Some participants were uncertain about how to utilise the feedback. Some participants suggested that if the feedback was more detailed, it would be more beneficial:

*‘Maybe just because it is now numerical, self-improvement cannot be based on it. It only gives the impression that I need to be vaguely better.’* (A1)

The feedback was considered trustworthy after simple consultations. However, some participants expressed doubts about its reliability if the consultation was complex. Furthermore, some participants experienced a conflict between the feedback and their own expectations. When receiving feedback, some participants experienced more positive or negative results compared with their expectations:

*‘But sometimes I get the feeling that I don’t always fully understand what exactly makes an encounter feel successful. Like, I might feel that the interaction went well, that the person felt seen and heard — but then it turns out it wasn’t actually received that well, or maybe the consultation was a bit of a flop, yet the patient is satisfied with it. So it’s not clear what should be done differently.’* (A8)

### Factors influencing PEI-Q2

The participants reflected on and expressed interest in multiple factors that could influence patient enablement. The participants considered PCPs’ personal characteristics, work experience, and workload as factors influencing patient enablement. The participants were interested in how to enhance patient enablement. PCPs’ actions during the consultation were seen as influential in improving patient enablement:

*‘The entire consultation is, in a way, an event where one can influence* [patient enablement] *in many ways. How much you listen to the patient, how much you let them explain the issue themselves, how you approach them, how you examine them, conduct the essential tests, and ensure they understand and know what the next steps are, whether they have accepted it, and if we agree on the matter and what to do next. This can probably have a significant impact.’* (B2)

Patients' expectations and characteristics, and indeterministic selection of patients for appointments were also regarded as important influencers of PEI-Q2 outcomes. Some participants expressed doubt about whether a sense of enablement could emerge immediately after an appointment. They suggested that the evolution of patient enablement sometimes requires time. This could impact feedback outcomes, especially with patients with chronic medical conditions:

*‘On the other hand, quite often patients have chronic, long-term issues, and are elderly, so I don't believe that a single consultation can significantly change or improve their quality of life. Because usually, there are long-term problems, and it is difficult to find a decisive factor.’* (B1)

In addition, participants suggested that organisational factors, such as how health centres operate, could impact the feedback. Some participants considered that patients might provide different feedback after visiting out-of-hours services. They also noted that the feedback might vary between face-to-face and remote consultations. The participants deliberated on whether continuity of care can have a positive effect on patient enablement. Some participants anticipated that a sense of enablement cannot be established during a short consultation:

*‘I feel that in an emergency situation, the patient might get the impression that the doctor is better because it is an acute situation.’* (B1)

*‘Among those treated remotely, it* [patient enablement] *is important information, as there is a constant emphasis on providing care mainly remotely.’* (A1)

### The use of PEI-Q2 results as a tool for comparison

The participants viewed the PEI-Q2 feedback as a potential tool for comparison between health centres, especially for managers. Comparing PEI results between health centres could enhance benchmarking to find the best practices for patient enablement:

*‘I don't know if comparing different health centers gives us individuals anything, but I thought maybe it would be useful for higher authorities. For example, if there are many people there who respond that they are the same or worse, what is being done differently there compared to where people respond that they are much better?’* (A2)

Opinions about comparing individual PCPs were divided among the participants. Some participants expressed concerns that comparisons could increase pressure and competitiveness. Some perceived that comparison does not necessarily contribute to personal development. However, some participants felt that the results were meaningless without comparison between peers. Furthermore, participants felt that comparing the results to one’s own previous performance would be beneficial:

*‘If the goal is to modify or develop one’s own actions, does comparing it to something else help me become better?’* (A8)

### Future development suggestions for the PEI-Q2

During the interviews, some participants suggested improvements to the PEI-Q2 feedback. They suggested that the feedback results could be categorised based on the type or reason for the consultation. Additionally, some participants requested the ability to link the feedback results with specific patients. Open feedback was also anticipated to complement the PEI results.

## Discussion

### Summary

Participating PCPs perceived patient enablement as an essential concept in primary health care. The PEI-Q2 feedback encouraged and motivated them, and they requested that it be made regular. However, the PEI-Q2 feedback was perceived to be rather difficult to interpret. PCPs expressed a willingness to understand how feelings of enablement develop. Opinions on comparing results at the health centre and personal levels were divided. PCPs suggested several forms of supplementary information that could improve the utility of the PEI-Q2 feedback.

### Strengths and limitations

To our knowledge, this is the first study to explore PCPs’ perceptions of the PEI-Q2 measurement. This study provides information on the acceptance and usability of the PEI-Q2 as a feedback tool. In the interviews, the participants reflected on the information that was represented in the educational intervention and extended the discussion to their own experiences and impressions about the PEI-Q2 and feedback.

However, this study has some limitations. The participating PCPs had only a brief experience of the PEI-Q2, and thus the discussion in the interviews represented the participants’ first impressions. Furthermore, the participants were not used to receiving regular, personal feedback. The educational events before the interviews may have affected the topics discussed in the interviews. In addition, the fact that four of the interviewees participated through Microsoft Teams may have affected the discussion. Also, the disparity between number of male and female participants may have influenced the topics discussed.

### Comparison with existing literature

The participating PCPs perceived patient enablement to be an important concept. This aligns with the core value of patient-centredness in general practice.^18^ In a previous qualitative study, PCPs identified the six-item PEI as a relevant tool for both patients and their care.^15^ However, some PCPs participating in our study were uncertain about how to utilise the PEI-Q2 results. There is limited knowledge on PEI-Q2 use, but the meaning and practical applications of numeric patient feedback were questioned by physicians in one previous study.^19^

Participating PCPs regarded PEI-Q2 as a more comprehensive measure compared with NPS. In previous studies, PEI has been compared with other PROMs and patient satisfaction measures. To some extent, PEI has an association to patient satisfaction measures.^20,21^ However, it seems to differ from other PROMs.^22^ Therefore, it might be beneficial to use PEI alongside other patient satisfaction measures and PROMs.

The participants expressed the need to understand the underlying factors contributing to the PEI-Q2 results. According to previous literature, there are numerous factors influencing patient enablement at the patient, GP, and practice levels.^23–33^ The participants were mainly interested in the same factors that have been studied before, but they were also interested in whether online appointments or the urgency of appointments had an effect on patient enablement.

The participants recognised PCPs’ actions in the practice as an important factor in evolving patient enablement. This is in concordance with previous studies that have shown that the length of consultation, the patient’s partnership with the doctor, the GP’s consultation skills, the GP’s person-centred approach, and patient involvement can have an effect on patient enablement.^24,26,28–32^

The comparison of individual PEI-Q2 results divided opinions. Comparison with one’s previous results was widely accepted, but some participants were doubtful about the comparison with peers. Accordingly, previous research indicates that feedback was perceived as more constructive when it did not include peer comparison.^34^ On the other hand, another study reported that physicians required peer comparison and specifically expected comparison for the same kind of patient population or area.^35^

The study participants saw the PEI-Q2 as a promising benchmarking tool between healthcare centres. To our knowledge, there are no previous studies exploring the use of the PEI or PEI-Q2 as a benchmarking tool. A previous study showed that PROMs were mainly used for clinical management.^36^ Furthermore, the relationship between benchmarking and quality improvement was examined in a recent review.^37^ The authors did not specifically study PROMs as a benchmarking tool, but they did show that benchmarking has the potential for quality improvement on the general level.^37^

Some participating PCPs stated that the PEI-Q2 was not necessarily useful on its own. A previous review discussed the importance of the narrative component in feedback,^5^ which is often lacking in numerical feedback. This highlights the need to complement quantitative tools, such as the PEI-Q2, with qualitative feedback to provide a meaningful evaluation.

### Implications for research and practice

This study provides valuable insights from PCPs’ into the implementation of the PEI-Q2 in practice and identifies key factors to consider. If the PEI-Q2 is being used for physicians’ feedback, it is essential to ensure that the recipients understand the factors influencing the measure’s results. Thus, adequate education considering patient enablement should be provided along with the implementation of the measure. In addition, providing open feedback or including some characteristics about the patients would enhance the recipients’ ability to effectively utilise the feedback.

Because this study presents the first impressions of receiving PEI-Q2 feedback, it would be beneficial to study this topic further with physicians who receive regular PEI-Q2 feedback. When delivering feedback, it is important to discuss the level of comparison with the recipients, as comparing them with their peers may induce stress. One future research direction could be to examine how physicians’ opinions vary with the use of the PEI-Q2 feedback comparing individuals, health centres, or wellbeing services counties. Furthermore, it would be beneficial to study patients’ perspectives of giving feedback with PEI-Q2.

In conclusion, the PEI-Q2 appears to be a promising tool for providing feedback. However, the numeric value of the PEI-Q2 could be insufficient for utilisation. With complementary information, such as open feedback or patient data, the feedback from the PEI-Q2 could be better accepted and utilised. In addition, professionals should be provided with education about patient enablement as part of the implementation process.

## Data

The dataset relied on in this article is available from the corresponding author on reasonable request.
